# Dominant drivers of spatiotemporal variations in carbon and water use efficiency across the Yellow River Basin revealed by interpretable machine learning

**DOI:** 10.3389/fpls.2025.1632172

**Published:** 2025-12-03

**Authors:** Guangchao Li, Wenjie Hao, Liqin Han, Mengjia Feng, Yanjie Li, Zhaoqin Yi, Yayan Lu, Kangjia Zuo

**Affiliations:** 1 College of Geography and Tourism, Henan Normal University, Xinxiang, China; 2 College of Life Sciences, Henan Normal University, Xinxiang, China

**Keywords:** water use efficiency, carbon use efficiency, Yellow River Basin, leaf area index, spatiotemporal heterogeneity, temperature

## Abstract

Precisely quantifying the spatiotemporal variation patterns of ecosystem water use efficiency (WUE) (i.e., WUE_NPP_ and WUE_GPP_) and carbon use efficiency (CUE) across diverse regions, as well as identifying the spatial heterogeneity of their principal influencing factors, are crucial for elucidating the complex underlying mechanisms governing carbon and water cycles in the Yellow River Basin (YRB). In this study, we utilized multi-source remote sensing data, and employed Ensemble Empirical Mode Decomposition (EEMD) to explore the nonlinear spatiotemporal trends and patterns of WUE_NPP_, WUE_GPP_, and CUE within the YRB ecosystem. Additionally, we applied the optimally parameterized XGBoost and SHAP models to discern the spatial heterogeneity of the key factors driving their spatiotemporal variations. The results showed that: (1) The WUE_NPP_, WUE_GPP_, and CUE of the YRB ecosystem exhibited a spatial distribution pattern characterized by higher values in the southeast and lower values in the northwest, with these metrics were predominantly concentrated at elevations ranging from 1000 to 1500 meters. (2) The interannual change rates of the yearly average values of WUE_NPP_, WUE_GPP_ and CUE in the YRB ecosystem were 0.008 ${\text{g C m}}^{-2}{\text{ mm}}^{-1}{\text{y}}^{-1}{\text{ a}}^{-1}$, 0.005 ${\text{g C m}}^{-2}{\text{ mm}}^{-1}{\text{y}}^{-1}{\text{ a}}^{-1}$, and 0.001, respectively. The predominant change patterns for WUE_NPP_ and WUE_GPP_ were monotonic increases, covering approximately 42.44% and 41.97% of the watershed area, respectively. In contrast, the change pattern for CUE was primarily a decrease followed by an increase, observed across 42.51% of the watershed area. (3) In the YRB ecosystem, the leaf area index (LAI) emerged as the primary determinant of WUE_NPP_ and WUE_GPP_. Specifically, WUE_NPP_ and WUE_GPP_ both showed an upward trend in tandem with the increase in LAI. Furthermore, temperature was identified as the key driving factor for CUE within the YRB ecosystem. (4) In the YRB ecosystem, LAI exhibited the highest importance index for both WUE_NPP_ and WUE_GPP_. It played a dominant role in approximately 42.80% and 45.35% of the study areas for WUE_NPP_ and WUE_GPP_, respectively. Conversely, temperature was a crucial factor influencing the spatial variability of CUE in the YRB ecosystem, exerting a predominant influence in 38.88% of the study areas.

## 1 Introduction

In the context of accelerating global warming, terrestrial ecosystems are undergoing profound structural and functional transformations, giving rise to complex environmental and ecological challenges such as carbon cycle imbalances, water scarcity, and biodiversity decline (Shao et al., 2024). To evaluate the stability of ecosystems under climate change, increasing attention has been directed toward carbon and water use efficiency (CWUE), a critical indicator of coupled carbon–water processes. CWUE reflects the ability of ecosystems to coordinate carbon fixation and water consumption under changing environmental conditions (Ding et al., 2021; Gao et al., 2016). It primarily consists of carbon use efficiency (CUE) and water use efficiency (WUE), the latter of which is further divided into WUE based on net primary productivity (WUE_NPP_) and WUE based on gross primary productivity (WUE_GPP_) (Jin et al., 2023). Collectively, these metrics characterize vegetation strategies in carbon acquisition and allocation per unit of water consumption, providing essential measures for assessing ecosystem adaptability and functional resilience (Ganjurjav et al., 2022).

The development of remote sensing technology has enabled large-scale, long-term monitoring of CWUE dynamics, making it a fundamental approach for investigating ecosystem responses to climate change (Cai et al., 2023; Liu et al., 2024b). CUE, a key variable in evaluating carbon sequestration efficiency, indicates the effectiveness of photosynthetic products being converted into biomass (Chakraborty et al., 2023). WUE, in contrast, highlights the trade-off between carbon uptake and water loss through evapotranspiration, serving as a central metric for understanding ecosystem functioning under global change (Kim et al., 2021). Typically, WUE_GPP_ is defined as the ratio of gross primary production (GPP) to evapotranspiration (ET), while WUE_NPP_ is defined as the ratio of net primary productivity (NPP) to ET (Ito and Inatomi, 2012).

Globally, extensive research has examined the spatiotemporal variability of CUE and WUE across diverse ecosystems. At the global scale, terrestrial CUE displays spatial patterns strongly associated with GPP and NPP, showing marked latitudinal variation, particularly between 30°N and 30°S (Gang et al., 2022). In contrast, WUE_GPP_ generally decreases from lower to higher latitudes in Central and East Asia (Kim et al., 2021; Wei et al., 2019). Investigations in specific ecosystem types, such as temperate forests and arid regions, further demonstrate coordinated variations between WUE and productivity. For instance, in the coniferous forests of Changbai Mountain, both WUE_NPP_ and NPP increased with forest age from 2000 to 2014. Similarly, natural vegetation in arid Northwest China experienced a shift from improvement to degradation in WUE_NPP_ and NPP between 2001 and 2018 (Li et al., 2019). Additional comprehensive assessments of CUE and WUE_GPP_ trends have been conducted in ecologically sensitive areas such as the karst regions of China (Xiao et al., 2023).

The spatiotemporal heterogeneity of WUE_GPP_ is shaped by multiple climatic, biological, and environmental factors (Wang et al., 2020) including drought (Liu et al., 2020a), aerosols (Lu et al., 2017), and human activities (Li et al., 2021). For example, in the mountainous areas of North China, leaf area index (LAI), temperature, and precipitation collectively explain 79.43% of the variation in WUE_NPP_ (Wang et al., 2023). On the Loess Plateau, the dominant drivers of WUE_NPP_ exhibit a latitudinal zonation, with precipitation and drought indices playing the primary roles, suggesting that water availability, rather than temperature, is the critical determinant in this region (Tian et al., 2020). Ecosystem sensitivities to environmental drivers also vary. For example, differences in respiration characteristics and GPP responses to temperature and humidity between natural forests and urban plantations indicate that CUE is primarily regulated by temperature and soil moisture (Li et al., 2022; Niu et al., 2011). Although elevated CO_2_ concentrations can enhance CUE (Liu et al., 2019), such positive effects may be diminished or even offset by climatic stressors such as drought and extreme heat (Mathias and Trugman, 2022).

The study area, located within the Yellow River Basin (YRB), spans multiple climate zones and vegetation types and is characterized by complex terrain, fragile ecosystems, and intensive human influence. In recent decades, the region has undergone substantial changes in temperature and precipitation patterns, coupled with large-scale afforestation, agricultural expansion, and urbanization. These factors jointly shape the carbon–water dynamics of local ecosystems, making the basin an ideal setting for examining ecosystem responses under combined natural and anthropogenic pressures.

Although previous studies have employed linear methods to investigate the spatiotemporal variations of ecosystem CWUE in the YRB and analyzed its relationships with climatic factors through correlation and contribution analyses, these approaches remain limited. For example, Li et al. (2025) applied linear regression and demonstrated that approximately 70.39% of the YRB exhibited a significant increasing trend in WUE_GPP_ from 1982 to 2018. Partial correlation analysis further indicated that precipitation (37.98%) and soil moisture (10.30%) were the dominant climatic drivers. Similarly, Tang et al. (2025) used linear regression to show that WUE_NPP_ and CUE declined in about 70% and 60% of the YRB, respectively, during 2001–2023, while relative contribution analysis revealed that annual mean temperature was negatively correlated with WUE_NPP_ in nearly 89% of the region and with CUE in about 74%. However, these studies largely rely on linear and correlative approaches, which are insufficient for capturing the nonlinear spatiotemporal patterns of CWUE, particularly regarding how its heterogeneous dynamics respond to human activities, vegetation changes, and climate variability.

In this context, the YRB were selected as the study area. Multi-source remote sensing data were combined with advanced analytical techniques, including ensemble empirical mode decomposition (EEMD), the XGBoost model, 10-fold cross-validation, stochastic search-based hyperparameter optimization, and SHAP interpretation. The objective of this study is to systematically characterize the nonlinear spatiotemporal dynamics of CWUE and to determine its dominant driving factors. The specific research objectives are as follows: (1) To uncover the features of spatial distribution characteristics, nonlinear spatiotemporal variation trends and patterns of CWUE in the YRB, while exploring the stability and sustainability of each variation pattern across different regions within the basin. (2) To elucidate the influence of driving factors (including climate change, vegetation change, and human activities) on the CWUE within the YRB; (3) To analyze the spatiotemporal heterogeneity of these driving factors across various regions of the YRB, with an emphasis on identifying the predominant factors that influence CWUE in different areas. This research aims to provide strategic guidance for the protection and rational use of natural resources within the YRB. It will also contribute to advancing the green development goals of the YRB, thereby promoting ecological conservation and sustainable development.

## 2 Study area

**Figure 1:**
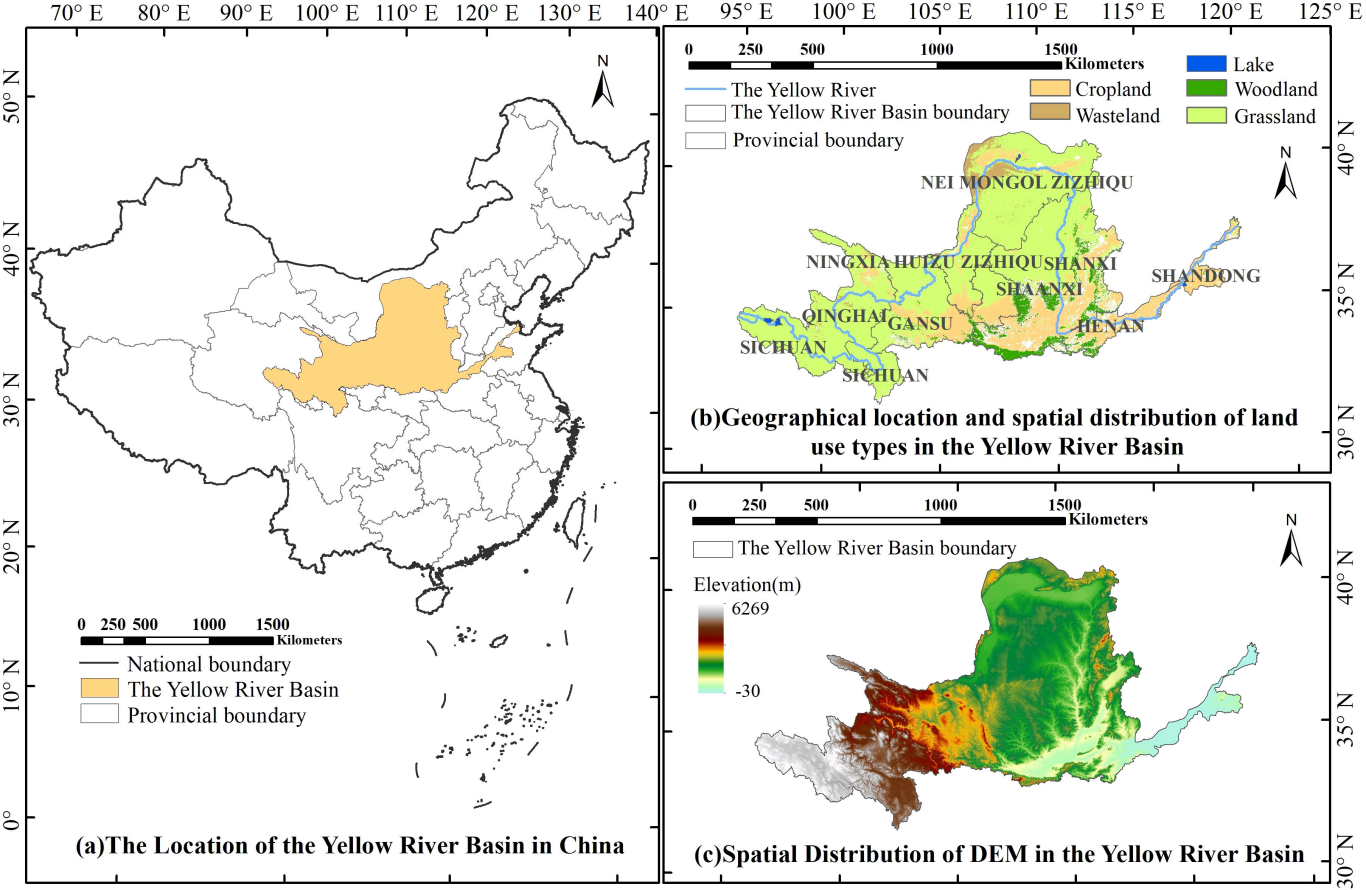
Study area. [**(a)** is the location of the Yellow River Basin in China, **(b)** is the spatial distribution of land use types in the Yellow River Basin, and **(c)** is the spatial distribution of DEM in the Yellow River Basin)].

The YRB encompasses the geographic and ecological regions traversed by the Yellow River, from its source to its estuary. The YRB is situated in northern and northwestern China, located at 32° N-42° N, 96° E-119° E (**Figure 1a**). Originating in the Bayan Har Mountains of Qinghai Province, the Yellow River flows through 9 provinces and 54 cities, including Qinghai, Sichuan, Gansu, Ningxia, Inner Mongolia, Shaanxi, Shanxi, Henan, and Shandong, before ultimately discharging into the Bohai Sea. The predominant land-use types within the YRB include grassland, cropland, woodland, and wasteland, which account for approximately 69.46%, 21.89%, 5.43%, and 3.22% of the total watershed area, respectively. Grasslands are primarily found in the middle and upper sections of the YRB, while woodlands are mainly concentrated in the southeastern portion of the basin (**Figure 1b**). Stretching from west to east, the YRB crosses four major geomorphologic units, namely the Tibetan Plateau, the Inner Mongolian Plateau, the Loess Plateau, and the Yellow-Huaihai Plain. The region is characterized by a general west-high and east-low topography (**Figure 1c**).

## 3 Research data and methods

### 3.1 Data

In the present study, the GPP data derived from a vortex-related data-light utilization efficiency model, which is based on vortex-related data-light utilization efficiency theory (Yuan et al., 2010, 2007). The latest version of the GPP product integrates components such as the photosynthetically active radiation absorption ratio (radiation) and atmospheric water vapor pressure, providing an accurate estimate of GPP. Furthermore, the NPP data used in this study exhibit a high level of accuracy (Li et al., 2023). Compared to the LAI data from the Moderate Resolution Imaging Spectroradiometer (MODIS), the LAI data from the GLASS dataset contain a larger proportion of high-quality data and show smoother temporal variations in their mapping (Jin et al., 2017). The ET data are generated using a Bayesian method combined with five latent heat flux algorithms. By incorporating MODIS and other reanalysis data, high spatiotemporal resolution and high-precision remote sensing data on heat fluxes are produced, covering the continuous latent heat surface space across the global land area. The radiometric data show no missing values. Even in areas with cloud cover, they maintain high quality. These data present continuous, consistent time-series curves, effectively reflecting the seasonal fluctuations in vegetation. Moreover, they exhibit excellent spatiotemporal consistency, thereby accurately capturing the dynamic changes in the vegetation over time and across different spatial locations. GLASS products undergo a rigorous spatial quality validation procedure to ensure high data accuracy (Zhao et al., 2013).

The sunlight data used in this study is based on observations from 824 benchmark and basic meteorological stations across mainland China. Spatial interpolation of these observations was performed using the thin-plate spline method. The interpolated data were then subjected to a threshold test against actual measured data from the meteorological stations, ensuring high data accuracy. A detailed description of all datasets used in this study is provided in **Table 1**.

**Table 1:** Summary of data used in this study.

Dataset	Unit	Time period	Spatial resolutions	Temporal resolutions	Data source
GPP	gCm^-2^y^-1^	1982-2018	5km	8 days	https://www.geodata.cn
NPP	gCm^-2^y^-1^	1982-2018	5km	8 days	https://www.geodata.cn
ET	mmy^-1^	1982-2018	5km	8 days	https://www.geodata.cn
LAI	N/A	2000-2018	5km	8 days	https://www.geodata.cn
Radiation	N/A	2000-2018	1km	8 days	https://www.geodata.cn/
Temperature	°C	2000-2018	1km	monthly scale	https://data.tpdc.ac.cn/
Precipitation	mm	2000-2018	1km	monthly scale	https://data.tpdc.ac.cn/
Sunlight	h	2000-2018	1km	annual scale	https://www.geodata.cn/
GDP	Million USD/km²	2000-2018	1km	annual scale	https://doi.org/10.6084/m9.figshare.17004523.v1
DEM	m	2000	30m	N/A	https://www.gscloud.cn/

Data preprocessing in this study involved several steps. Initially, raw GPP, NPP, and ET data were converted from HDF format to raster format, followed by batch extraction and mosaicking. ArcPy was then used to crop the raster images to the study area (YRB) and resample them to a consistent spatial resolution. Next, ArcPy was employed to calculate CWUE values at the pixel level across the YRB. Finally, the vector-scale TIFF image of the YRB was cropped and resampled using ArcPy to ensure spatial resolution consistency.

### 3.2 Methods

#### 3.2.1 Carbon and water use efficiency

CWUE consists of three components: (i) CUE, which is defined as the ratio of NPP to GPP, (ii) WUE_GPP_, calculated as the ratio of GPP to ET (Yang et al., 2020), and (iii) WUE_NPP_, determined as the ratio of NPP to ET (Tian et al., 2010).

#### 3.2.2 EEMD model

The EEMD model was employed to analyze the nonlinear variation trends and patterns of CWUE in the YRB. This model effectively captures nonlinear components within statistical trends without relying on prior assumptions. Its data decomposition is based on local characteristics, resulting in enhanced time-frequency resolution and clearer physical meaning (Yin et al., 2016). Consequently, the EEMD model has been widely adopted in studies involving long-time-series remote sensing data analysis (Wen et al., 2017). The computational steps for applying EEMD are presented as follows:

The new signal, X(t), to be decomposed, is obtained by adding Gaussian white noise of varying amplitudes to the original signal, x(t):

(1) X(t)=x(t)+w(t)

In Equation 1, w(t) represents the added Gaussian white noise.

Compute the average value of the upper and lower envelopes $\varphi_{1}(t)$ in Equation 2:

(2) $$X_{1}(t)=X(t)-\varphi_{1}(t)$$

Based on the standard deviation (SD), assess whether the process is capable of continuing:

(3) $$SD=\sum_{t=0}^{t}\left[❘X_{i}(t)-X_{i-1}(t){❘}^{2}/X_{\left(i-1\right)}^{2}(t)\right]$$

In Equation 3 i represents the iteration count. If this value is smaller than the preset threshold, halt the above calculation procedure and compute the first iteration result for variable IMF1 in Equation 4:

(4) $$IMF1=X_{j}(t)=X_{j-1}(t)-\varphi_{j}(t)$$

Calculate the residuals of the signal $X_{1}(t)$ and IMF1:

(5) $$R(t)=X_{1}(t)-IMF1$$

Repeat Equations 1 and 5 until $R_{n}(t)$ turns into a monotonic function in Equation 6:

(6) $$R_{i}=R_{i-1}-IMF_{i},i=2,3,\ldots ,n$$

According to the steps described above, the number of residuals and the value of IMFs separated by x(t) can be obtained as shown in Equation 7:

(7) $$x(t)=\sum_{i=1}^{n}IMF_{i}(t)+R(t)$$

Based on the monotonicity of the eigenvalue trend and the characterization of the extreme points, the change patterns were classified into five categories: monotonically increasing, monotonically decreasing, increasing followed by decreasing, decreasing followed by increasing, and those that did not meet the significance criteria for variation.

#### 3.2.3 Hurst index

The Hurst index (*H*) was calculated using the rescaled range ( R/S) analysis to assess the inter-annual sustainability of the CWUE. The specific formula for this calculation can be found in the relevant literature (Khosroshahi et al., 2023). The *H* value domain ranges from 0 to 1. When 0 <*H*< 0.5, it indicates inverse persistence in the CWUE change, meaning that the future trend of CWUE variation is opposite to its past trend. When *H* = 0.5, it suggests that the time series of CWUE time series is random, with the future trend of CWUE being difficult to predict. When 0.5 <*H*< 1, it indicates sustainability in CWUE changes implying that the past trend of CWUE will persist, and the future trend will align with the previous trend. The sustainability of different CWUE change patterns in the YRB was assessed by combining the results of the EEMD model and the Hurst exponent, with detailed information provided in **Table 2**.

**Table 2:** Sustainability of different variation patterns of CWUE in the YRB.

*H* value	Variation patterns of CWUE	Sustainability of different variation patterns of CWUE
>0.5	Monotonically increasing	Sustainability and monotonically increasing
>0.5	Monotonically decreasing	Sustainability and monotonically decreasing
>0.5	Increasing then decreasing	Sustainability and increase then decrease
>0.5	Decreasing then increasing	Sustainability and decrease then increase
<0.5	Failed to pass significant change	Undetermined future variation trend

#### 3.2.4 Coefficient of variation

The coefficient of variation (CV) was used to quantify the magnitude of inter-annual fluctuations in CWUE. The detailed calculation methodology is provided in a previous study (Zhang et al., 2016). In this study, the values of the CV values were categorized into three classes to represent the stability of different CWUE variation patterns across the YRB. Specifically, high volatility was defined as CV ≥ 0.3, medium volatility as 0.1 < CV < 0.3, and low volatility as CV ≤ 0.1. This classification was performed by integrating the EEMD model with the CV, allowing for a comprehensive evaluation of CWUE stability. Further details can be found in **Table 3**.

**Table 3:** Stability of different variation patterns of CWUE in the YRB.

$CV_{CWUE}$	Variation patterns of CWUE	Stability of different patterns of change in CWUE	Grade
$CV_{CWUE}\geq 0.3$	Monotonically increasing	High fluctuation and monotonically increasing	XLI
	Monotonically decreasing	High fluctuation and monotonically decreasing	XXXI
	Increasing then decreasing	High fluctuation and increasing then decreasing	XXI
	Decreasing then increasing	High fluctuation and decreasing then increasing	XI
$0.1<CV_{CWUE}<0.3$	Monotonically increasing	Medium fluctuation and monotonically increasing	XLIII
	Monotonically decreasing	Medium fluctuation and monotonically decreasing	XXXIII
	Increasing then decreasing	Medium fluctuation with increasing and then decreasing	XIII
	Decreasing then increasing	Medium fluctuation, decreasing then increasing	XXIII
$CV_{CWUE}\leq 0.1$	Monotonically increasing	Low fluctuation and monotonically increasing	XLII
	Monotonically decreasing	Low fluctuation and monotonically decreasing	XXXII
	Increasing then decreasing	Low fluctuation and increasing then decreasing	XII
	Decreasing then increasing	Low fluctuation and decreasing then increasing	XXII

#### 3.2.5 Optimal parameters XGBoost and SHAP models

##### 3.2.5.1 Optimal parameterized XGBoost model

The XGBoost (eXtreme Gradient Boosting) model is an advanced implementation of the gradient boosting framework, renowned for its execution speed and predictive performance (Niazkar et al., 2024). It operates by constructing an ensemble of weak learners (typically decision trees) in a sequential, additive manner. Each new tree is trained to correct the residuals or errors of the combined previous ensemble. This iterative process effectively reduces bias and mitigates underfitting. The core principle of XGBoost lies in its objective function, which incorporates a loss function and a regularization term to control model complexity and prevent overfitting as shown in Equation 8:

(8) $$Obj(\theta )=\sum_{i=1}^{n}l(y_{i},{\hat{y}}_{i})+\sum_{k=1}^{K}\Omega (f_{k})$$

**Figure 2:**
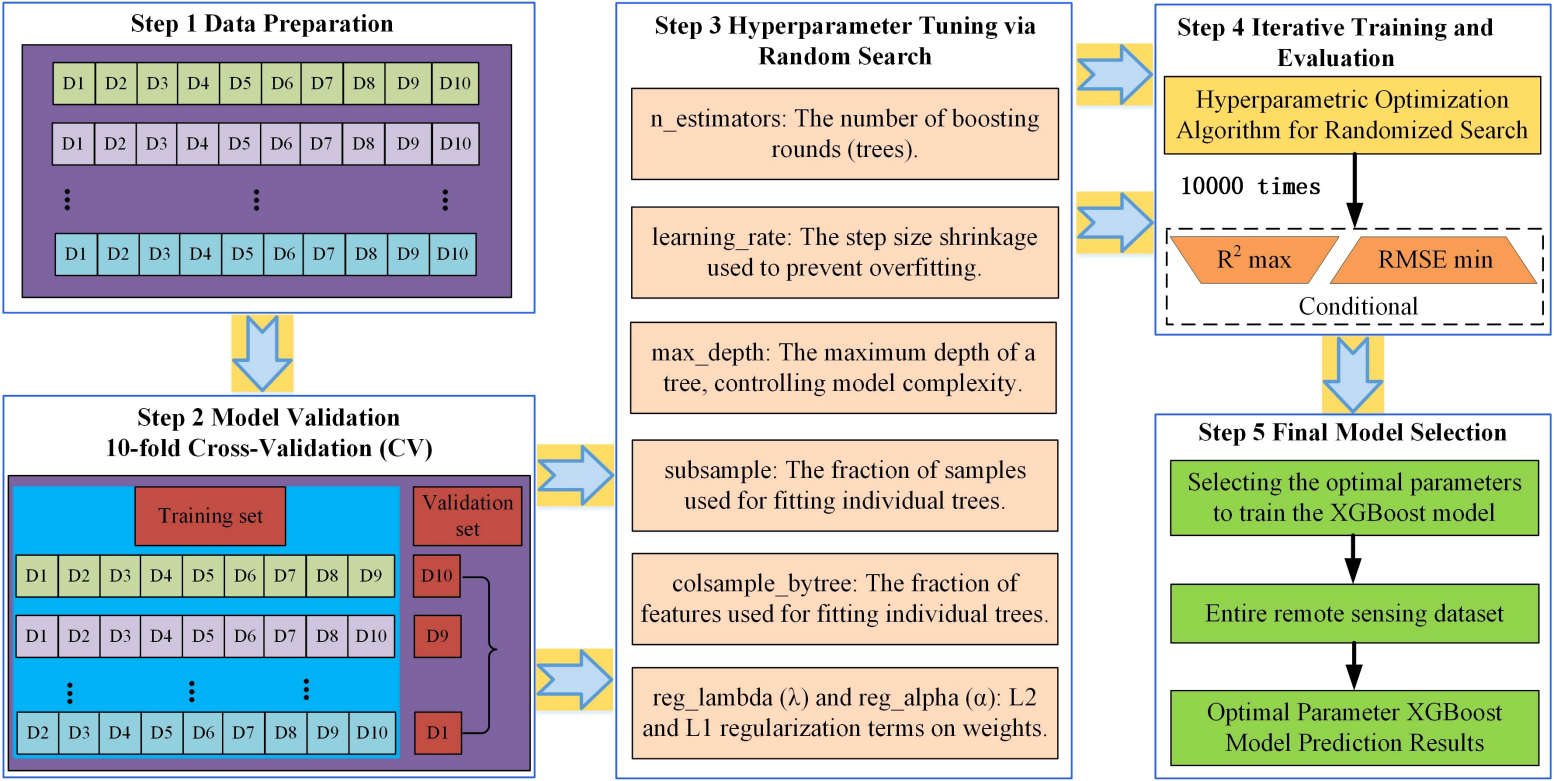
Working principle of the optimal parameter XGBoost model.

Where $y_{i}$ and ${\hat{y}}_{i}$ are the observed and predicted values of the i-th sample, respectively; l is a differentiable convex loss function (e.g., mean squared error for regression); $\Omega (f_{k})=\gamma T+1/2\lambda ∥w{∥}^{2}$ is the regularization term for the k-th tree, with T being the number of leaves, w being the leaf weights, and γ and λ being regularization parameters that penalize the complexity of the tree. Model training, validation, and hyperparameter optimization steps:

Step 1: Data preparation: The dataset containing CWUE indicators and their potential driving factors was partitioned into a training set (70%) and a testing set (30%).
Step 2: Model validation-10-fold Cross-Validation (CV): To ensure robustness and avoid overfitting, the training set was further subjected to 10-fold CV. The dataset was randomly split into 10 equal-sized subsets. The model was trained 10 times, each time using 9 subsets for training and the remaining 1 subset for validation. The average performance across all 10 folds was computed to evaluate the model’s generalizability.
Step 3: Hyperparameter tuning via random search: To enhance predictive accuracy, a random search strategy was employed for hyperparameter optimization. This method randomly samples a predefined number of combinations from a wide hyperparameter space, which is more efficient than grid search for high-dimensional spaces. The key hyperparameters tuned included: n_estimators: The number of boosting rounds (trees). Learning_rate: The step size shrinkage used to prevent overfitting. Max_depth: The maximum depth of a tree, controlling model complexity. Subsample: The fraction of samples used for fitting individual trees. Colsample_bytree: The fraction of features used for fitting individual trees. Reg_lambda (λ) and reg_alpha (α): L2 and L1 regularization terms on weights.
Step 4: Iterative training and evaluation: The XGBoost model underwent 10,000 training iterations with randomly sampled hyperparameter combinations. The performance of each model was evaluated using the Root Mean Square Error (RMSE) and the Coefficient of Determination (R²) on the validation sets from the CV process.
Step 5: Final model selection: The hyperparameter set that yielded the lowest average RMSE (or highest R²) across the CV folds was selected to train the final model on the entire training set. The framework of this optimization process is illustrated in **Figure 2**. GPU acceleration and parallel processing were leveraged to expedite this computationally intensive process.

**Table 4** presents the optimal hyperparameters for the three CWUE indicators in the YRB derived from this research. All models achieved a *R*^2^ exceeding 0.87, demonstrating high goodness of fit. These results validate the optimal parameter XGBoost model effectively and reliably captures the spatiotemporal heterogeneity of the key drivers influencing CWUE dynamics in the YRB.

**Table 4:** Optimal hyperparameter details for optimal parameter XGBoost.

CWUE	N estimators	Learning rate	Max depth	Subsample	R^2^	RMSE
CUE	294	0.1399	9	0.8973	0.8781	0.0735
WUE_NPP_	423	0.1206	9	0.7243	0.9510	0.0728
WUE_GPP_	423	0.1206	9	0.7243	0.9813	0.0787

##### 3.2.5.2 SHAP explanatory model

The SHAP method, grounded in game-theoretic principles, offers a systematic approach to quantify the individual contributions of features to a model’s prediction. Characterized by properties such as local accuracy, tolerance to missing values, and consistency, SHAP values are computed using the following procedure:

(9) $$x_{i}=\sum_{s\in S_{i}}\frac{(n-❘s❘)!(❘s❘-1)!}{n!}[v(s)-v(s/i)]$$

In Equation 9, $x_{i}$ is the set containing all subsets of member i, s is some set case of $S_{i}$, ❘s❘ indicates the amount of elements of the subset s, n refers to the number of collaborators, v(s) is the gain of the set s. v(s/i) signifies the gain of the set excluding member i.

The SHAP value offers a reliable metric for quantifying the individual contributions of each driver to the final predicted outcome of the model.

(10) $$y_{i}=y_{base}+f(x_{i,1})+f(x_{i,2})+\ldots +f(x_{i,j})+\ldots +f(x_{i,k})$$

In Equation 10, $y_{i}$ is the predicted value of the $i_{th}$ sample, $y_{base}$ is the predicted mean value of all samples, $x_{i}$ is the $i_{th}$ sample, $f(x_{i,j})$ is the SHAP value of the $i_{th}$ sample, $j_{th}$ feature, and k is the number of input features.

In this study, the SHAP explanatory model was applied to quantify the contributions of the following driving factors to the spatiotemporal variations of CWUE in the YRB: temperature, precipitation, sunlight, radiation, LAI, and GDP. These factors represent key climatic, biological, and anthropogenic influences on ecosystem CWUE.

## 4 Results

### 4.1 Spatial and vertical distribution of CWUE

#### 4.1.1 Spatial distribution characteristics of CWUE

**Figure 3:**
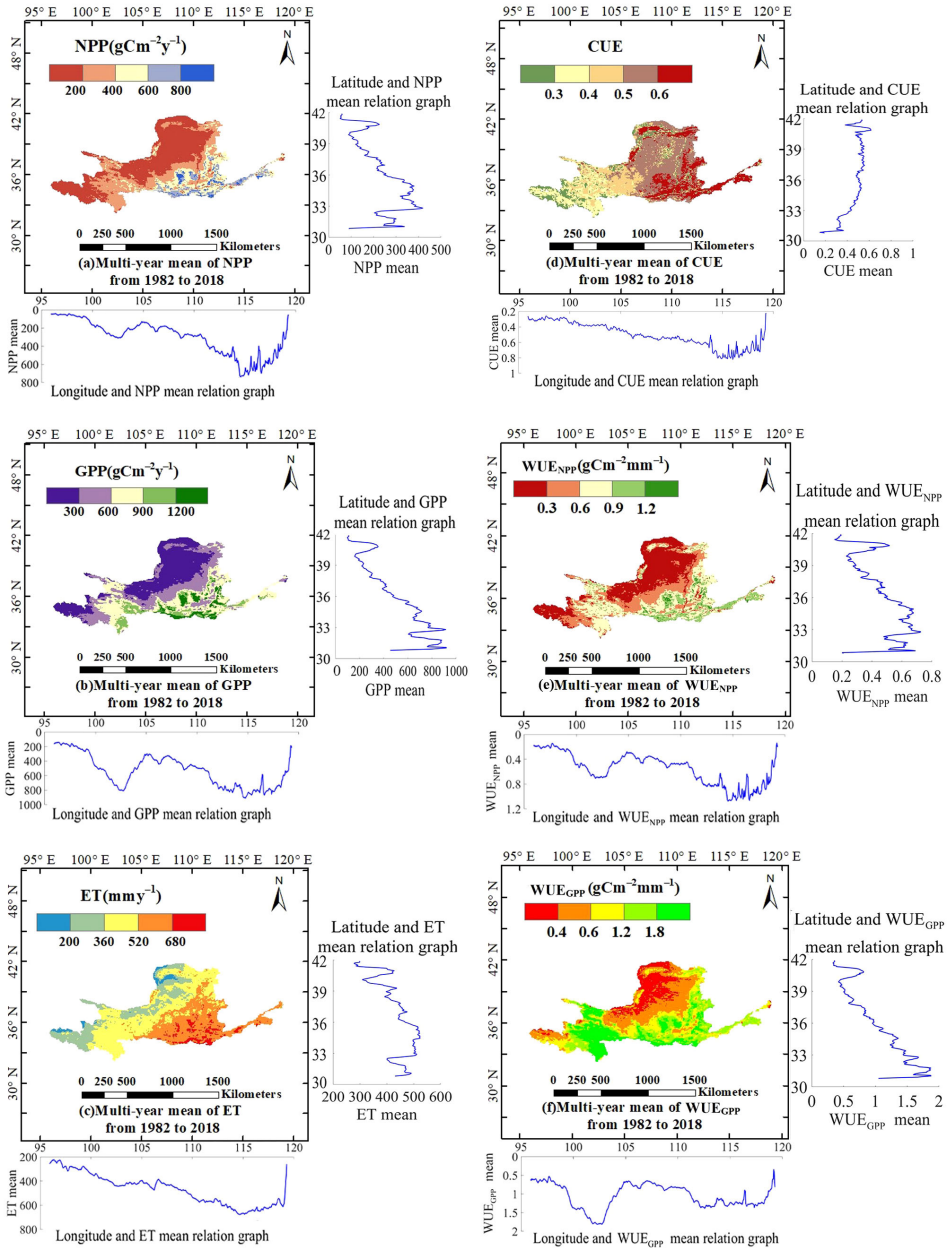
Spatial distribution of CWUE and its relationship with latitude and longitude in the YRB from 1982 to 2018. Specifically, **(a–f)** show the spatial distributions of the multi-year mean from 1982 to 2018 for NPP, GPP, ET, CUE, WUE_NPP_, and WUE_GPP_, respectively.

The spatial distribution of CWUE in the YRB from 1982 to 2018 and its relationship with latitude and longitude are shown in **Figure 3**. WUE_NPP_ and WUE_GPP_ in the YRB exhibit a spatial pattern characterized by higher values in the southeast and lower values in the northwest, with relatively elevated WUE_NPP_ and WUE_GPP_ observed at the junction of Qinghai and Gansu provinces. The spatial distributions of WUE_NPP_ and WUE_GPP_ closely resemble those of NPP and GPP, respectively, while GPP, NPP, and ET generally increase from the northwest to the southeast. In contrast, CUE shows a gradually increasing trend from west to east, with pronounced regional differences. In the southwestern part of the basin (the upper reaches in the southwest), the long-term averages of CWUE, ET, NPP, and GPP are comparatively low, whereas these indicators are relatively high in the middle and lower reaches of the basin. In terms of longitude, WUE_NPP_ and WUE_GPP_ in the YRB initially increase, then decrease, and subsequently follow a similar alternating pattern with further increases and decreases as longitude increases, while CUE first increases and then decreases with increasing longitude. Both exhibit a sharp decline around 115°E. In terms of latitude, WUE_NPP_ and WUE_GPP_ show a decreasing trend with increasing latitude, whereas CUE increases with latitude. The variation patterns of WUE_GPP_ and GPP with latitude in the YRB largely correspond to those of WUE_NPP_ and NPP, with both reaching their minimum values at 41.66°N and their maximum values occur at 31.03°N and 32.81°N, respectively.

#### 4.1.2 The variation of CWUE at different elevations

**Figure 4:**
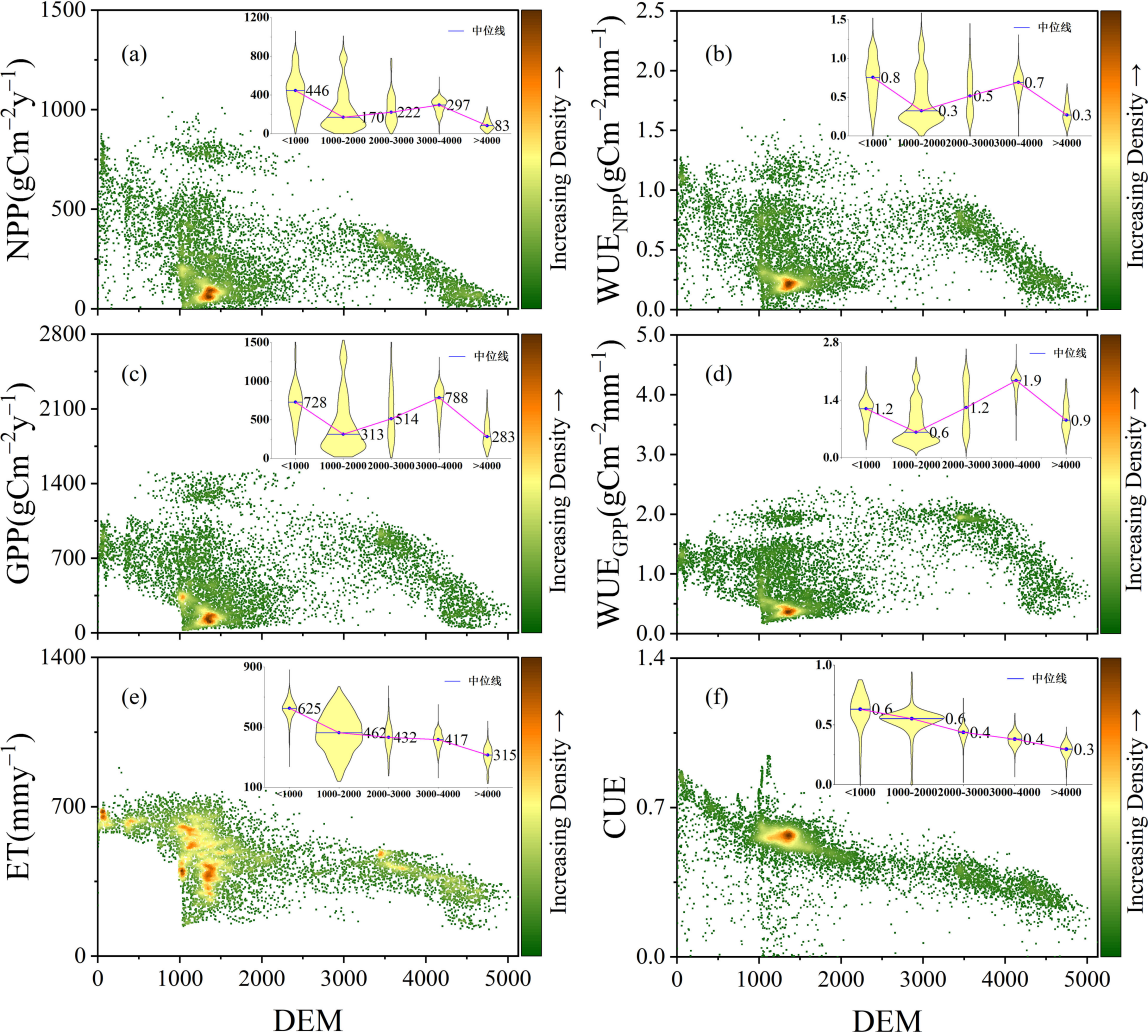
Distribution of CWUE at different elevations in the YRB from 1982 to 2018 [**(a** is the distribution of NPP at different altitudes, **b** is the distribution of WUE_NPP_ at different altitudes, **c** is the distribution of GPP at different altitudes, **d** is the distribution of WUE_GPP_ at different altitudes, **e** is the distribution of ET at different altitudes, and **f** is the distribution of CUE at different altitudes).

The distribution of CWUE at different elevations in the YRB from 1982 to 2018 is illustrated in **Figure 4**. WUE_NPP_ and WUE_GPP_ in the YRB exhibit a trend of initially decreasing, then increasing, and subsequently decreasing with elevation, with their turning points coinciding with those of NPP and GPP at approximately 3000–4000 m. In contrast, CUE decreases with increasing elevation. CWUE in the basin is primarily concentrated at elevations between 1000 and 1500 m, and WUE_NPP_ within this elevation range spans 0-1.5 ${\text{g C m}}^{-2}{\text{ mm}}^{-1}$. The values of WUE_NPP_, WUE_GPP_, and CUE are mainly within the ranges of 0.18-0.24 ${\text{g C m}}^{-2}{\text{ mm}}^{-1}$, 0.36-0.42 ${\text{g C m}}^{-2}{\text{ mm}}^{-1}$, and 0.55-0.59, respectively. The elevation-dependent variation trends of GPP and NPP with elevation are similar to those of WUE_NPP_ and WUE_GPP_. Additionally, the median values of WUE_GPP_ and GPP within each elevation interval exceed those of WUE_NPP_ and NPP. The elevational trend of ET is similar to that of CUE, with ET values not falling below 400 mmy^−1^ within the 0–1000 m elevation range.

### 4.2 Spatiotemporal dynamics of CWUE

#### 4.2.1 Interannual variation trend of CWUE

**Figure 5:**
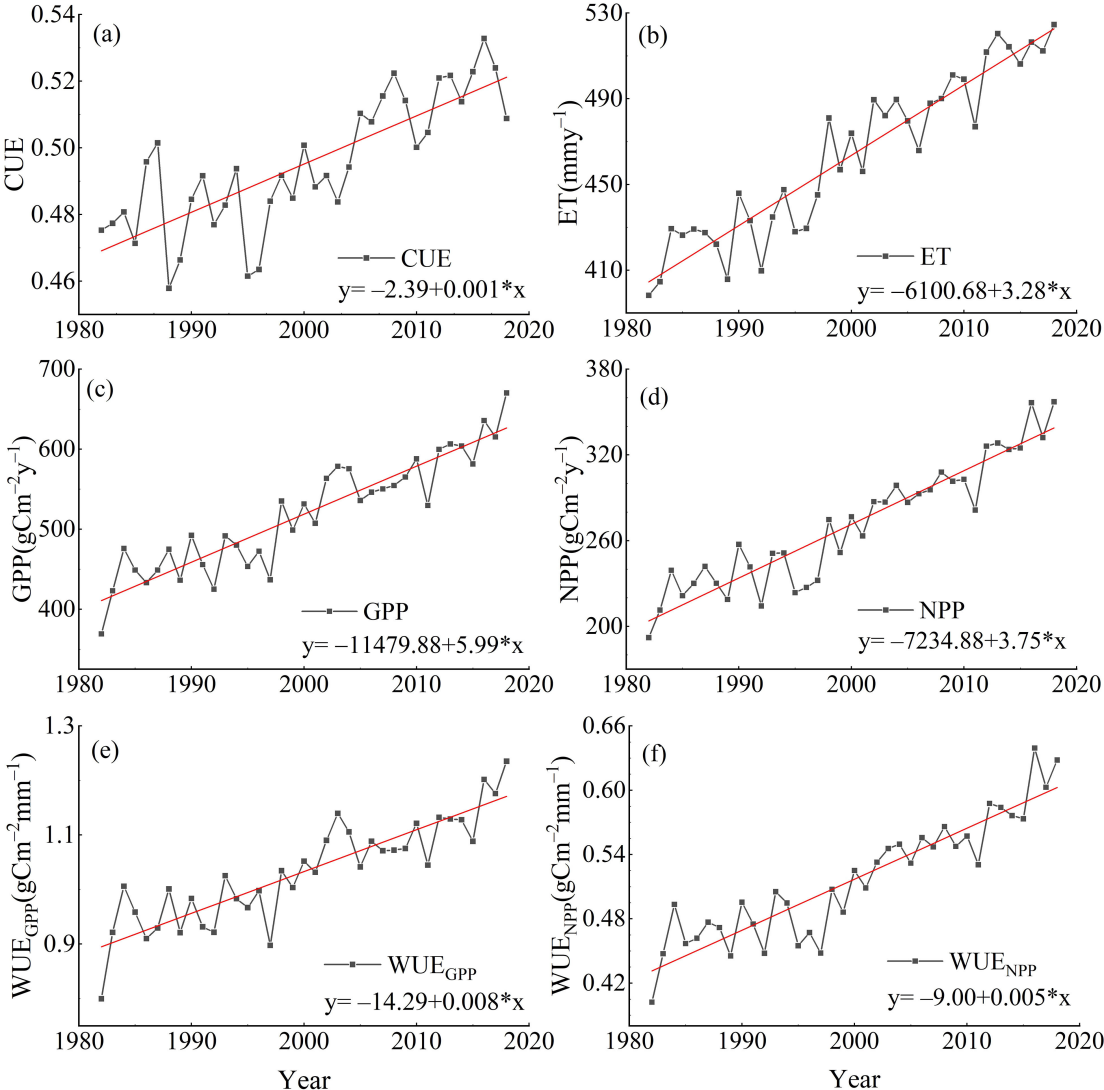
Interannual variation of the annual mean CWUE in the YRB from 1982 to 2018 [**(a)** is the interannual variation of annual average CUE, **(b)** is the interannual variation of annual average ET, **(c)** is the interannual variation of annual average GPP, **(d)** is the interannual variation of annual average NPP, **(e)** is the interannual variation of annual average WUE_GPP_, and **(f)** is the interannual variation of annual average WUE_NPP_)].

The interannual variation of the annual mean CWUE in the YRB from 1982 to 2018 is illustrated in **Figure 5**. Over this period, the annual mean CWUE in the YRB exhibited an increasing trend. The interannual variation rates for the annual mean WUE_NPP_, WUE_GPP_, and CUE were 0.008 ${\text{g C m}}^{-2}{\text{ mm}}^{-1}{\text{ a}}^{-1}$, 0.005 ${\text{g C m}}^{-2}{\text{ mm}}^{-1}{\text{ a}}^{-1}$, and 0.001, respectively, with their long-term averages being 0.52 ${\text{g C m}}^{-2}{\text{ mm}}^{-1}$, 1.03 ${\text{g C m}}^{-2}{\text{ mm}}^{-1}$, and 0.50. The average values over the long-term for NPP, GPP, and ET were 271.40 ${\text{g C m}}^{-2}{\text{ y}}^{-1}$, 518.73 ${\text{g C m}}^{-2}{\text{ y}}^{-1}$, and 463.59 ${\text{mm y}}^{-1}$, respectively. Each of these variables demonstrated an upward trend over the period from 1982 to 2018.

#### 4.2.2 Spatial variation trends and patterns of CWUE

**Figure 6:**
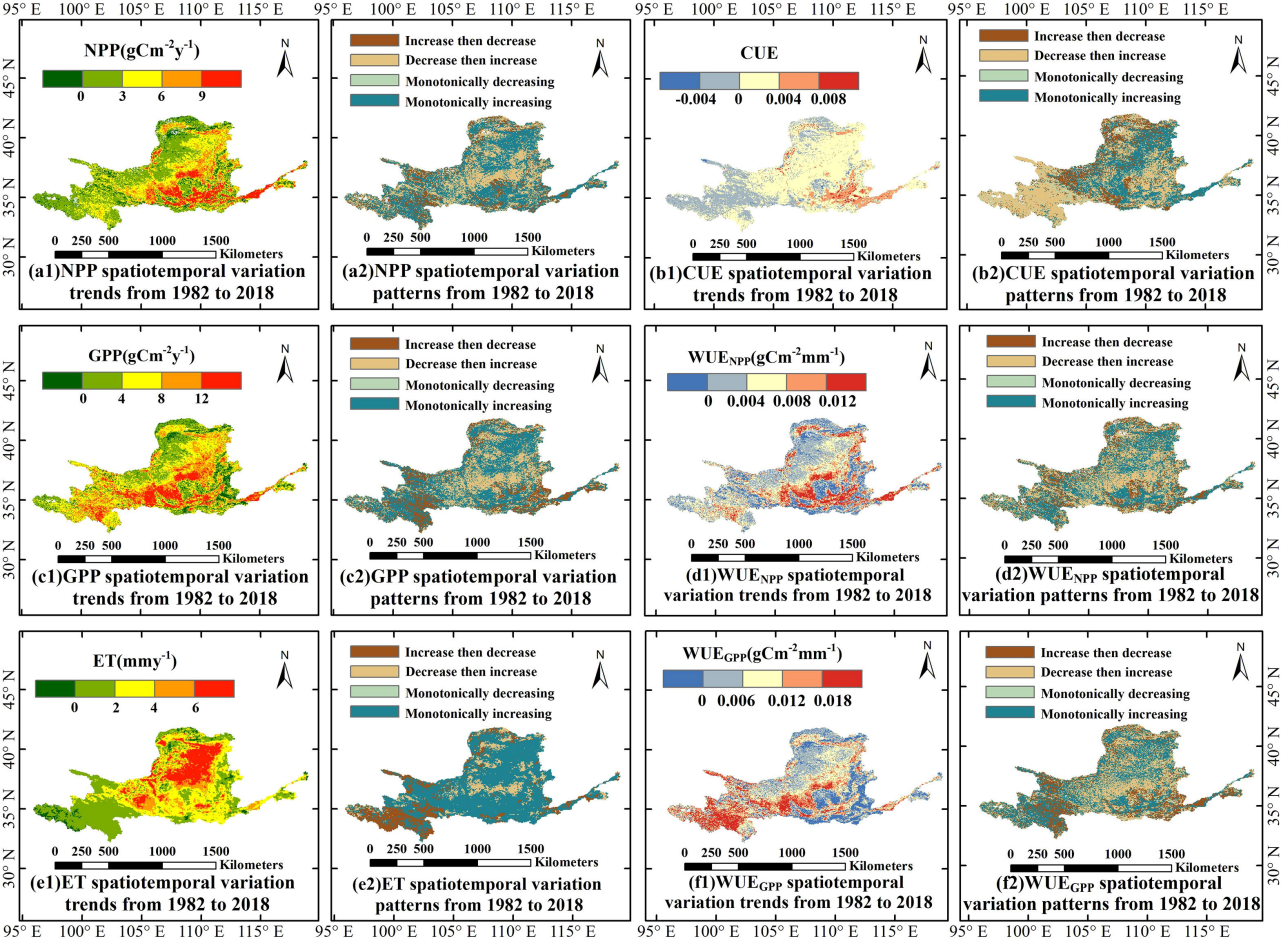
Variation trend and pattern of the annual mean CWUE in the YRB from 1982 to 2018. Specifically, **(a1, b1, c1, d1, e1, f1)** show the spatiotemporal variation trends from 1982 to 2018 for NPP, CUE, GPP, WUE_NPP_, ET, and WUE_GPP_, respectively; **(a2, b2, c2, d2, e2, f2)** show the spatiotemporal variation patterns from 1982 to 2018 for NPP, CUE, GPP, WUE_NPP_, ET, and WUE_GPP_, respectively.

**Figure 6** illustrates the variation trends and patterns of the annual mean CWUE in the YRB from 1982 to 2018. The variation patterns of WUE_NPP_ and WUE_GPP_ are closely aligned. The monotonically increasing variation pattern accounts for about 42.44% and 41.97% of the total area within the basin, respectively, while the “decrease then increase” pattern represents approximately 36.20% and 31.40%, respectively. These patterns are primarily concentrated in the middle section of the YRB’s reaches. In the central and lower reaches of the YRB, the monotonically increasing variation pattern of CUE is dominant, covering approximately 34.22% of the basin area. In contrast, approximately 42.51% of the upstream region exhibits a distinct “decrease then increase” pattern, primarily distributed in eastern Qinghai, northern Sichuan, and southwestern Gansu provinces. The significant increasing trend of CUE indicates that carbon sequestration efficiency is gradually improving across most areas of the YRB. Moreover, pixels showing monotonically decreasing trends in WUE_NPP_, WUE_GPP_, and CUE account for the smallest proportions of the basin, at 3.67%, 4.62%, and 6.18%, respectively, and are sparsely distributed in space. The variation patterns of NPP, GPP, WUE_NPP_, and WUE_GPP_ are largely consistent, with a predominant increasing trend. The monotonically increasing patterns of NPP and GPP cover about 51.55% and 51.53% of the overall area of the basin, respectively. NPP and GPP in the central region of YRB follow a pattern of “decrease then increase” in larger areas. ET in the YRB is predominantly characterized by a monotonically increasing trend, covering approximately 69.48% of the basin and widely distributed across regions except for the southwestern part. In fact, areas exhibiting an increasing ET trend account for as much as 96.52% of the basin, indicating that the rise in ET is nearly basin-wide. Overall, except for WUE_NPP_ and WUE_GPP_, other indicators show a relatively pronounced increasing trend in the middle and lower reaches of the basin, particularly in the lower reaches, whereas WUE_NPP_ and WUE_GPP_ exhibit minor decreasing trends in parts of the middle reaches.

#### 4.2.3 Sustainability and stability of CWUE

**Figure 7:**
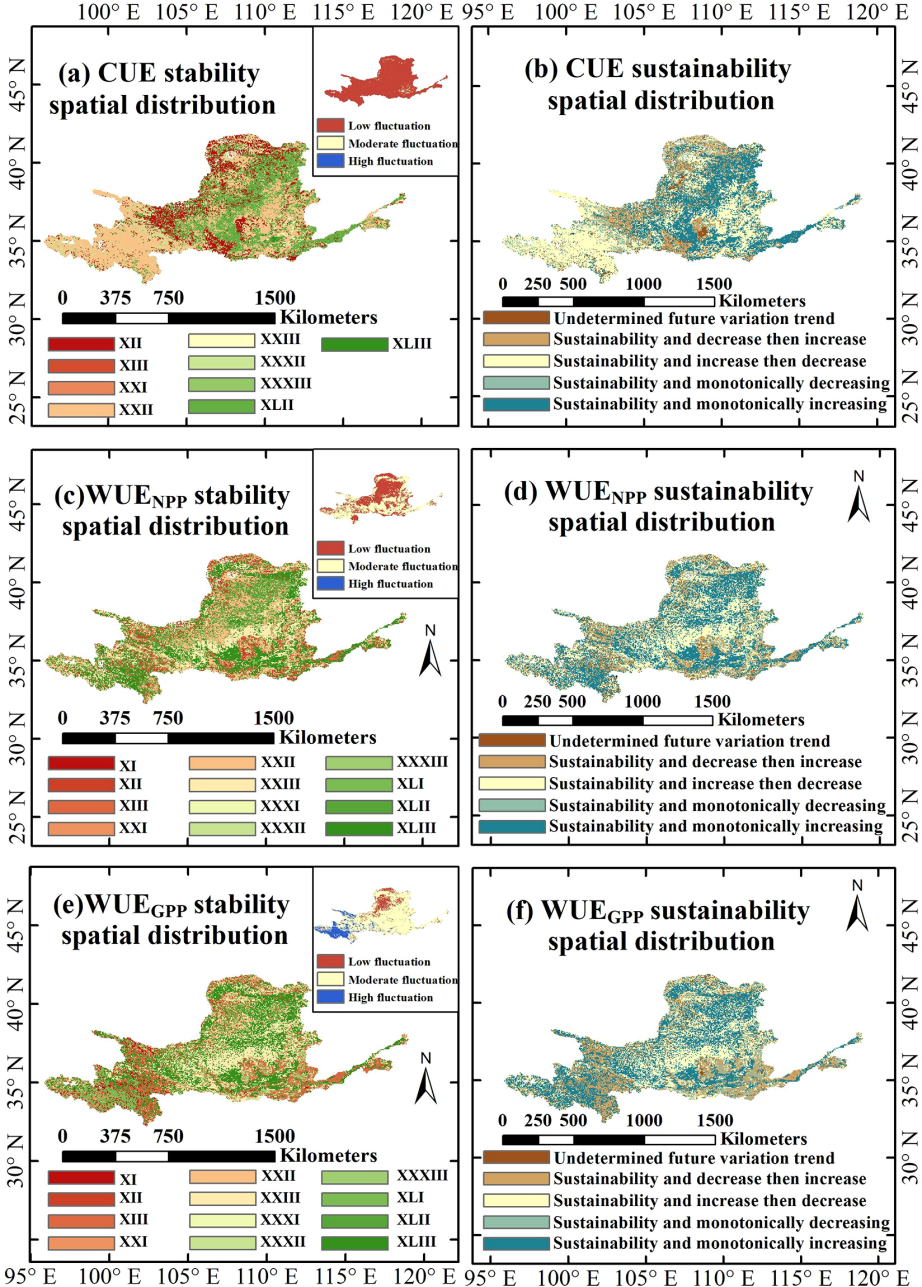
Spatial distribution of the sustainability and stability of CWUE in the YRB from 1982 to 2018. Specifically, **(a, c, e)** show the spatial distributions of stability for CUE, WUE_NPP_, and WUE_GPP_, respectively. **(b, d, f)** show the spatial distributions of sustainability for CUE, WUE_NPP_, and WUE_GPP_, respectively.

The spatial distribution of the sustainability and stability of CWUE in the YRB from 1982 to 2018 is illustrated in **Figure 7**. The sustainable spatial patterns of WUE_GPP_ and WUE_NPP_ in the YRB exhibit remarkable similarity, both predominantly showing sustainable and monotonically increasing trends, accounting for approximately 41.40% and 41.68% of the basin area, respectively, with more pronounced patterns in the lower reaches. In the middle reaches, both indicators mainly display sustainable trends of initially increasing followed by decreasing, covering approximately 30.46% and 35.03% of the area, respectively. CUE in the basin predominantly exhibits a sustainable pattern of initially increasing followed by decreasing, accounting for about 40.98% of the area, and is mainly distributed in the southwestern (upper reaches in the southwest) and eastern parts of the basin. Overall, CWUE in the YRB demonstrates considerable sustainability. Both WUE_NPP_ and WUE_GPP_ primarily show moderate fluctuations, accounting for approximately 52.88% and 73.41%, respectively. Additionally, both exhibit a “moderate fluctuation and monotonically increasing” variation pattern (XXXIII), accounting for about 25.47% and 29.80%, respectively. Moreover, WUE_GPP_ exhibits relatively high fluctuation in the southwestern part of the YRB (upper reaches in the southwest), whereas moderate fluctuation dominates in the middle and lower reaches, and low fluctuation is mainly concentrated in the northwest. For WUE_NPP_, low fluctuation is primarily observed in the northern regions of the basin, with the middle and lower reaches particularly the lower reaches also showing pronounced low fluctuation. CUE in the YRB is predominantly characterized by low fluctuation, accounting for approximately 97.91% of the total basin area. Among these, areas with low fluctuation and an initially decreasing followed by increasing pattern (XXII) cover about 41.44%, mainly distributed in the upper southwestern region, while areas with low fluctuation and monotonically increasing trends (XLII) account for approximately 33.37%, indicating that CUE has exhibited very high stability across the basin from 1982 to 2018.

### 4.3 Identification of driving factors for CWUE

#### 4.3.1 Trends and patterns of driving factors

**Figure 8:**
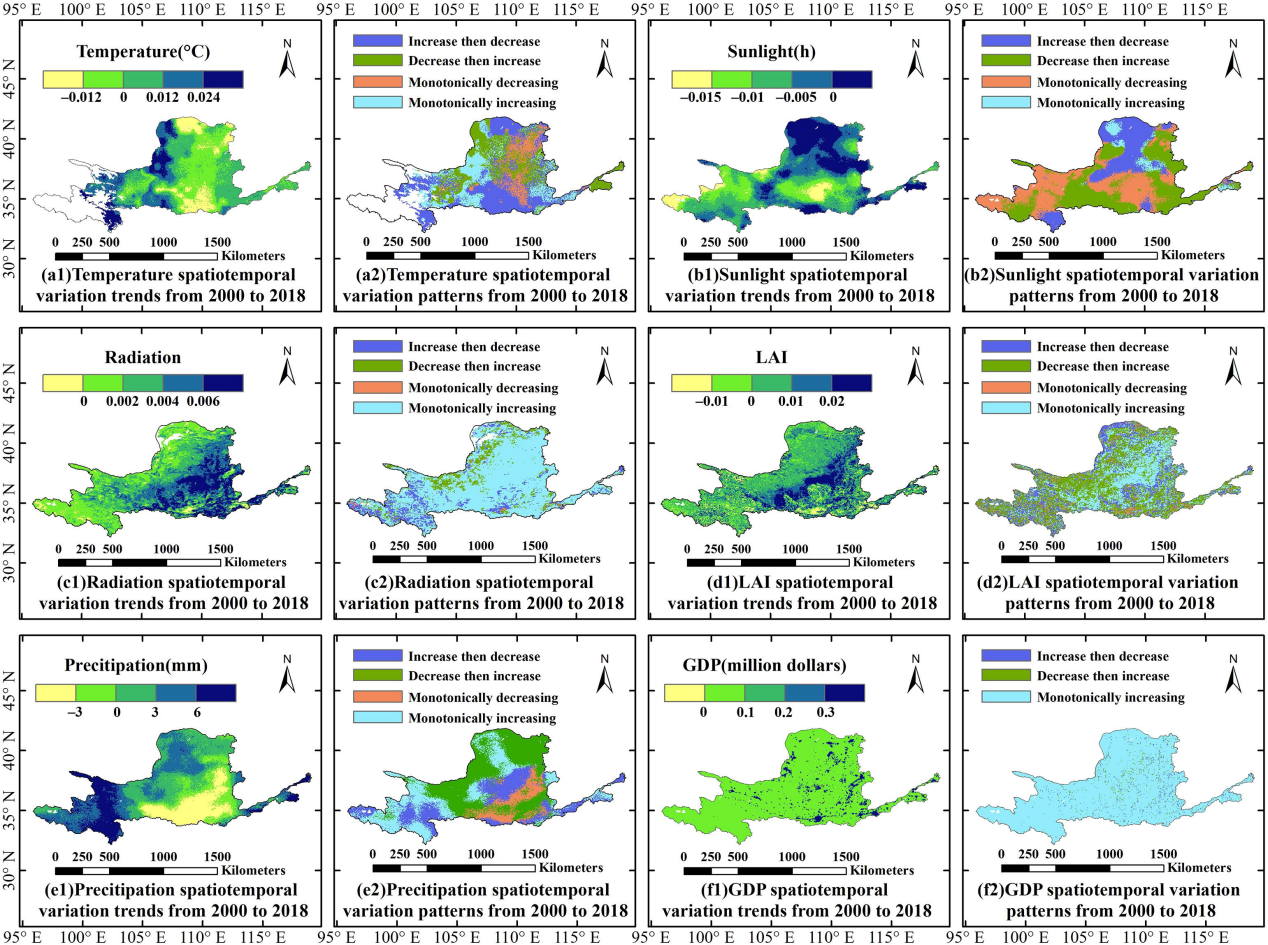
Trends and patterns of driving factors in the YRB from 2000 to 2018. Specifically, **(a1, b1, c1, d1, e1, f1)** show the spatiotemporal variation trends from 2000 to 2018 for Temperature, Sunlight, Radiation, LAI, Precipitation, and GDP, respectively; **(a2, b2, c2, d2, e2, f2)** show the spatiotemporal variation patterns from 2000 to 2018 for Temperature, Sunlight, Radiation, LAI, Precipitation, and GDP, respectively.

**Figure 8** illustrates the trends and patterns of driving factors in the YRB from 2000 to 2018. Sunlight in the YRB exhibits a significant decreasing trend (75.90%), while other driving factors show notable increasing trends. The proportion of areas exhibiting these trends, from largest to smallest, is as follows: GDP (99.85%), radiation (97.04%), LAI (79.02%), precipitation (70.60%), and temperature (57.39%). In most areas of the middle reaches of the YRB, temperature exhibits a decreasing trend, with the “increase then decrease” pattern accounting for approximately 39.29% of the basin, whereas temperature in the lower reaches predominantly shows an increasing trend. The rate of change in radiation displays a west-to-east increasing pattern (i.e., progressively increasing from the upper to the middle and then to the lower reaches), with the monotonically increasing pattern being dominant, covering about 75.40% of the basin. In the upper reaches of the YRB, the rate of precipitation change is relatively high, particularly in the southwestern part of the basin, while the “decrease then increase” pattern predominates in most areas of the middle reaches, accounting for approximately 39.71% of the basin. The rate of change in sunlight is relatively high in the northern regions, with the northwest primarily exhibiting an “increase then decrease” pattern, whereas the lower reaches mainly show a “decrease then increase” pattern. LAI is primarily characterized by the “decrease then increase” and “monotonically increasing” patterns, accounting for about 73.46%. GDP predominantly follows a “monotonically increasing” variation pattern, covering approximately 95.91% of the area. The increasing trends of both variables are widely distributed across the basin.

#### 4.3.2 Importance of CWUE driving factors

**Figure 9:**
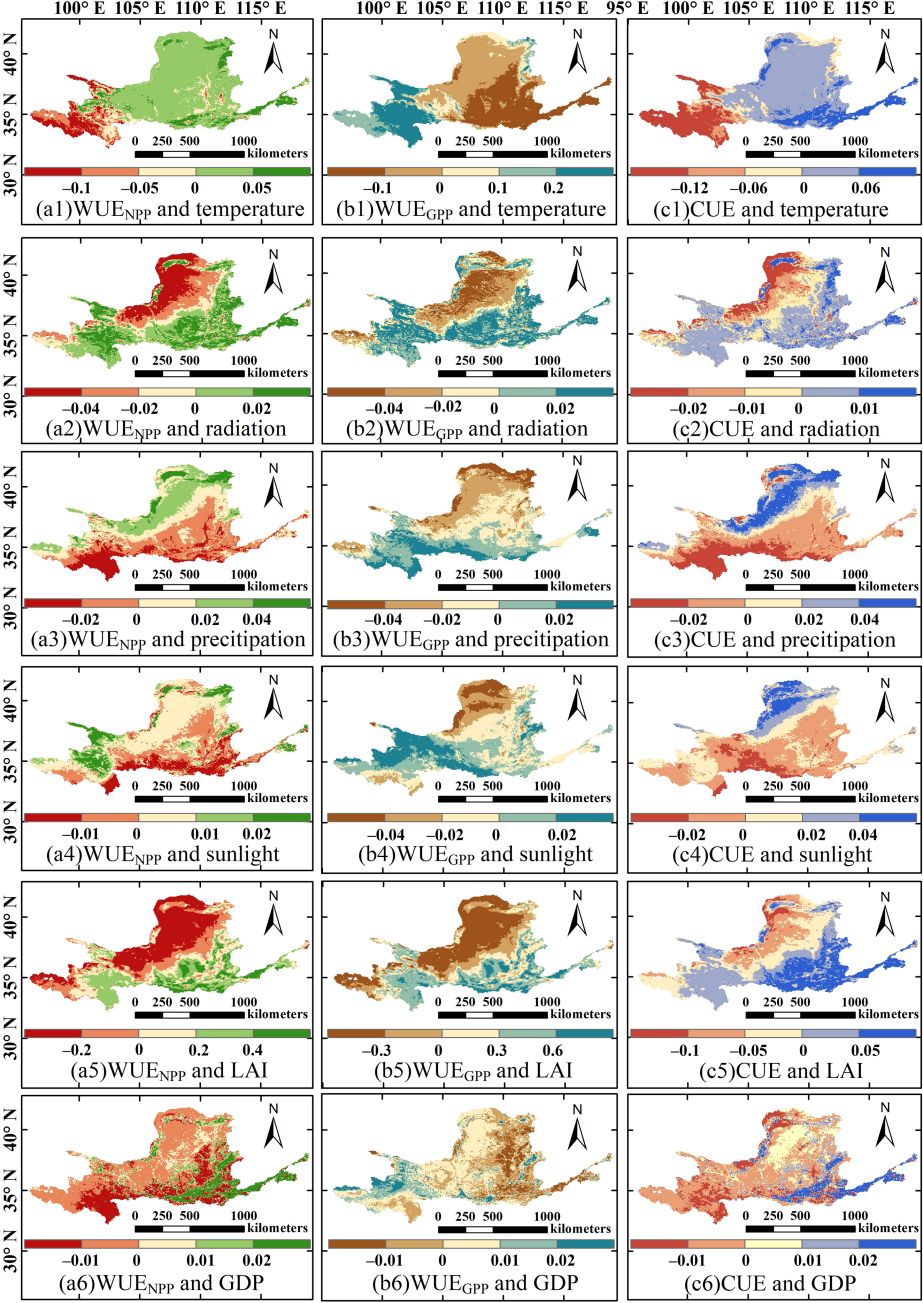
The spatial pattern of driving factors and SHAP values of CWUE in the YRB from 2000 to 2018. **(a1–6)** respectively shows the spatial pattern and SHAP value of WUE_NPP_ and driving factors (temperature, radiation, precipitation, sunlight, LAI, and GDP). **(b1–6)** respectively shows the spatial pattern and SHAP value of WUE_GPP_ and driving factors (temperature, radiation, precipitation, sunlight, LAI, and GDP). **(c1–6)** respectively shows the spatial pattern and SHAP value of CUE and driving factors (temperature, radiation, precipitation, sunlight, LAI, and GDP).

**Figure 9** illustrates the spatial pattern of driving factors and SHAP values of CWUE in the YRB from 2000 to 2018. Temperature and sunlight have a positive impact on the spatial variation of WUE_NPP_, accounting for 73.56% and 60.90% of the region, respectively. accounting for 73.56% and 60.90% of the region, respectively. This effect was mainly concentrated in the northwestern part of the YRB, whereas the negative influence of precipitation on WUE_NPP_ increased from north to south. The spatial distributions of the effects of radiation and LAI on WUE_NPP_ are relatively similar, with predominantly positive effects in the southeastern part of the YRB (middle and lower reaches) and predominantly negative effects in the northwest (northeastern part of the upper reaches). The negative effect of GDP on WUE_NPP_ covers most of the basin (67.22%), particularly in the upper reaches. In the northern part of the YRB, the driving factors primarily exert negative effects on WUE_GPP_. The negative impacts of temperature and precipitation on WUE_GPP_ were particularly prominent, accounting for approximately 62.50% and 60.73% of the YRB, respectively, with their effects being most evident in the mid-reaches of the YRB. Moreover, the negative effect of temperature on WUE_GPP_ is also relatively pronounced in the lower reaches. Radiation, LAI, and sunlight showed significant negative impacts on WUE_GPP_ in the northwestern part of the YRB (the northeastern part of the upper reaches) were relatively significant, while the positive effects were more dominant in the southern part. The absolute SHAP values of LAI were relatively large. In the mid-upper sections of the YRB, GDP predominantly exerted a positive influence on WUE_GPP_. This positive correlation is observed across about 59.40% of the basin’s area. The positive effects of temperature, radiation, and LAI on CUE are relatively pronounced, accounting for 67.23%, 53.26%, and 53.82% of the YRB, respectively, and are particularly prominent in the southeastern part of the basin (middle and lower reaches, especially the lower reaches). In contrast, precipitation, radiation, and GDP mainly exert negative effects on CUE, accounting for approximately 55.24%, 52.83%, and 60.29%, respectively. Among these, the negative effects of precipitation and radiation are more pronounced in the southern part of the basin (the southwestern upper reaches and the middle and lower reaches). The negative effect of GDP on CUE is more significant in the upper reaches of the basin, whereas in the lower reaches (southwestern part of the basin), GDP predominantly exerts a positive effect on CUE.

**Figure 10:**
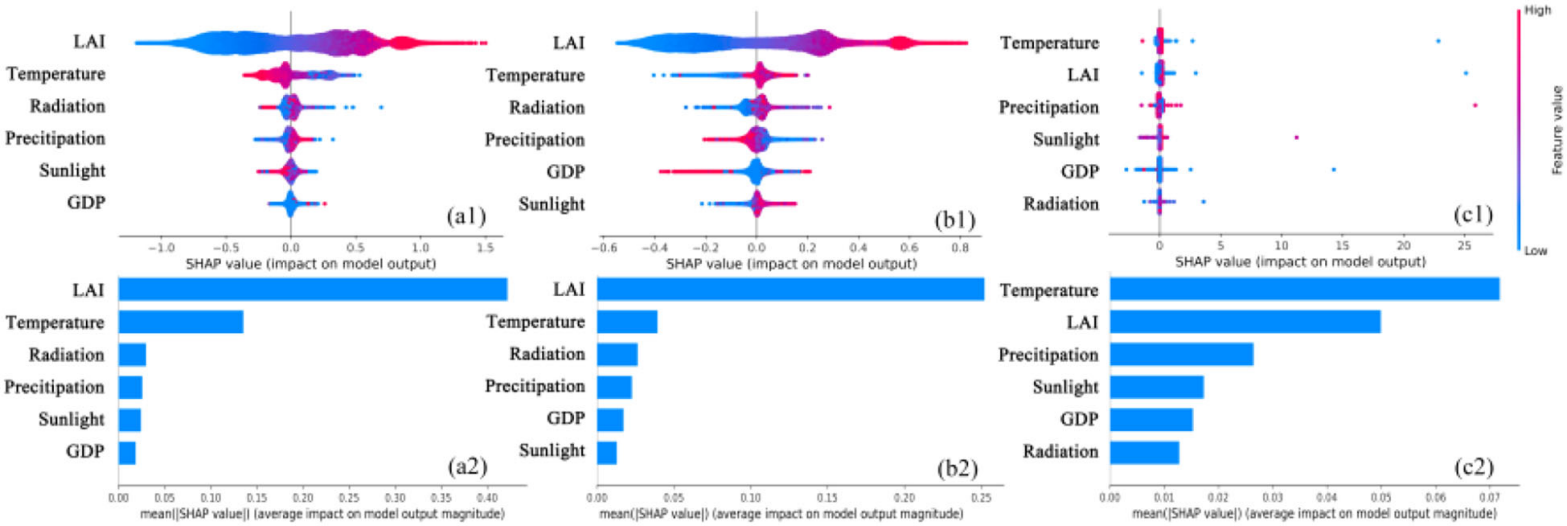
Summary of SHAP values of driving factors in the YRB (WUE_GPP_: **(a2)** WUE_NPP_: **(b2)** CUE: **(c2)**) and their relative importance to ecosystem CWUE (WUE_GPP_: **(a1)** WUE_NPP_: **(b1)** CUE: **(c1)**), 2000-2018. The magnitude of the eigenvalue is denoted by the color, while the density is represented by the vertical distribution.

**Figure 10** presents the summarized SHAP values of the driving factors and their relative importance to ecosystem CWUE in the YRB during the period from 2000 to 2018. In the YRB, LAI was identified as the primary driver of WUE_NPP_ and WUE_GPP_. As LAI increased, both WUE_NPP_ and WUE_GPP_ showed an upward trend. Temperature was the secondary driver, while a rise in temperature resulted in an increase in WUE_NPP_, it simultaneously caused a decrease in WUE_GPP_. In contrast, sunlight had the least impact on WUE_NPP_, and GDP had the smallest effect on WUE_GPP_ in the YRB. In contrast, sunlight and GDP respectively had the most marginal influence on WUE_NPP_ and WUE_GPP_ in the YRB. The driving factors influencing CUE, listed in descending order of their weights, were temperature, LAI, precipitation, sunlight, GDP, and radiation. LAI and temperature were determined to be the most influential driving factors for CWUE within the YRB ecosystem.

**Figure 11:**
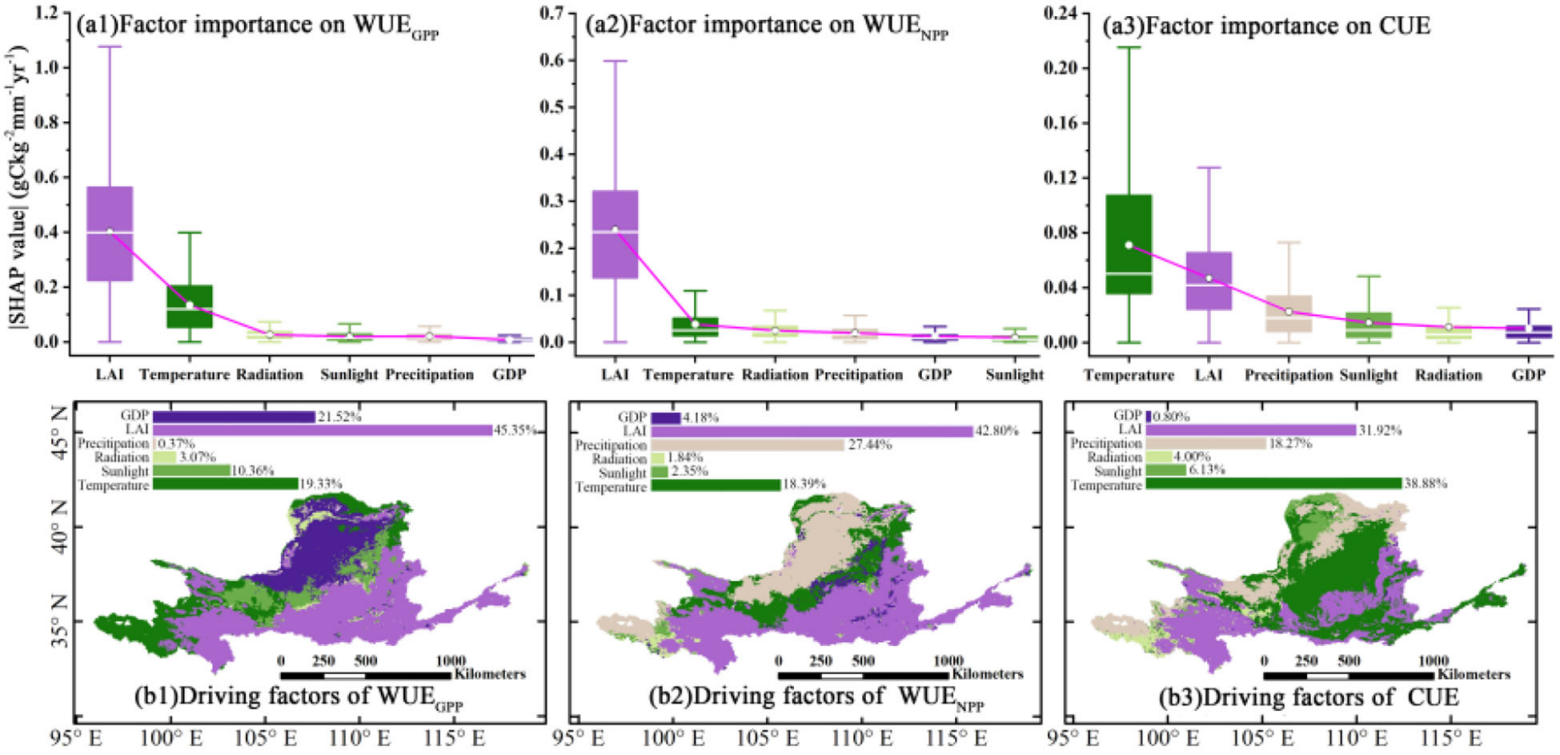
Spatial distribution of dominant factors and their importance index ranking of spatiotemporal changes of CWUE in the YRB ecosystem from 2000 to 2018. **(a1-3)** show the statistics of absolute SHAP values of influencing factors’ contribution to CWUE, with white circles indicating the mean values and pink lines connecting the magnitude of factor importance. Box plots show the statistics of the absolute values of SHAP for each factor, where **(a1-3)** exclude extreme values. **(b1-3)** show the main driving factors for the same image element that dominate the CWUE at each 0.05° × 0.05° point, and the histograms show what percentage each factor accounts for as a main driving force across the whole study area.

**Figure 11** illustrates the spatial distribution of the dominant factors contributing to the spatiotemporal variations in CWUE within the ecosystem of the YRB ecosystem from 2000 to 2018, along with their rankings based on importance indices. Among these factors, LAI exhibits the highest importance indices for WUE_NPP_ and WUE_GPP_, at approximately 0.24 ${\text{gCm}}^{-2}{\text{mm}}^{-1}{\text{yr}}^{-1}$ and 0.40 ${\text{gCm}}^{-2}{\text{mm}}^{-1}{\text{yr}}^{-1}$, respectively. It plays a dominant role in approximately 42.80% of the study area for WUE_NPP_ and 45.35% for WUE_GPP_. These areas primarily cover the southern region of the YRB, the southeastern part (the middle and lower reaches), as well as the junction between Qinghai and Gansu Provinces respectively. In the northwestern part of the YRB (the northeastern part of the upper reaches), precipitation holds the highest importance index for WUE_NPP_, while GDP shows the highest importance index for WUE_GPP_, dominating approximately 27.44% and 21.52% of the study areas respectively. Temperature significantly influences the spatial variation of CUE, with an importance index of approximately 0.05. It is the dominant factor in 38.88% of the study area, particularly pronounced in the lower reaches of the basin. LAI also significantly influences the spatial variation of CUE, with a dominant effect in 31.92% of the study area, particularly pronounced in the southern part of the YRB. Overall, temperature and LAI are the dominant factors in over 61.19% of the study areas, particularly in the mid-lower sections of the YRB. In contrast, radiation has a relatively minor impact on the spatial variation of CWUE.

## 5 Discussion

### 5.1 Spatial distribution of CWUE in the YRB

The spatial distribution of the multi-year average CWUE in the YRB exhibited distinct patterns across latitude, longitude, and elevation. The findings revealed that WUE_GPP_ tended to be higher in the eastern and southern parts of the YRB while it was lower in the western and northern parts. These results largely correspond to the findings of prior studies (Fan et al., 2023; Sun et al., 2022). In the YRB, WUE_GPP_ at the junction of Qinghai and Gansu Provinces is higher than in the surrounding areas (Liu et al., 2024a). The spatial distributions of WUE_NPP_ and WUE_GPP_ in the YRB are similar. Additionally, as demonstrated by Liu et al. (2015), the spatial distributions of NPP and ET exhibit an upward trend from the northwest region to the southeast region. The annual mean ET in the YRB is lower in the western and northern areas, which are characterized by sparse vegetation, and higher within the eastern and southern regions, where vegetation is more abundant. This pattern is in line with the dispersion in space of evapotranspiration in China as studied by previous researchers (Li et al., 2017). CUE is higher in the Loess Plateau, as noted by Liu et al. (2022). Its overall distribution exhibits lower values in the northwestern region, and this spatial pattern corresponds to the southeast-northwest hydrothermal gradient across the YRB (Chakraborty et al., 2023).

Between 1982 and 2018, spatial heterogeneity in CWUE was observed across different latitudes and longitudes in the YRB. The trends of WUE_GPP_, WUE_NPP_, GPP, and NPP across these regions were generally consistent, with a decrease observed as latitude increased. This aligns with the findings of Wei et al. (2019) and Kim et al. (2021), who reported that WUE_GPP_ showed a downward trend from south to north in the Central Asian and East Asian regions. A similar decreasing trend with increasing latitude was observed for ET, which corresponds to the gradient distribution of precipitation and vegetation cover, as described by Zhang et al. (2016). In contrast, CUE increased with latitude. Additionally, between 1982 and 2018 in the YRB, WUE_NPP_ and WUE_GPP_ in the YRB followed a pattern of increase, decrease, subsequent increase, and decrease with increasing longitude. CUE exhibited a characteristic pattern of increasing initially and then decreasing as longitude increased. As reported by Liang et al. (2015), NPP in the YRB exhibited a distinct increasing trend from west to east. This spatial pattern was primarily driven by climatic gradients, while variations in WUE and CUE indicated a trade-off between water availability limiting productivity and carbon allocation strategies adapted to regional temperature conditions.

### 5.2 Spatiotemporal variation of CWUE in the YRB

From 1982 to 2018, the CWUE indicators in the YRB generally exhibited a fluctuating upward trend. Specifically, WUE_NPP_ increased by a factor of 0.005 g C m^–2^ mm^–1^ a^–1^, which aligns well with the findings of Zhang et al. (2016). In the YRB, WUE_GPP_ grew at a rate of 0.008 g C m^–2^ mm^–1^ a^–1^, with an average multi-year value of 1.03g C m^–2^ mm^–1^, largely consistent with previous studies. For instance, Li et al. (2021) reported that the multi-year average of China’s WUE_GPP_ in China from 2001 to 2017 was 1.08gCm^–2^mm^–1^, with an annual increment of 0.003gCm^–2^mm^–1^a^–1^. The spatiotemporal variations in WUE_GPP_ are directly influenced by changes in ET and GPP. This finding aligns with previous research, such as by Liu et al. (2020b), which suggests that significant increases in global GPP and ET have a direct impact on WUE_GPP_. From 1982 to 2018, vegetation WUE_GPP_ increased significantly across most regions globally, as reported by Ji et al. (2021). The spatiotemporal variations observed in the YRB study area in our study generally corroborate these prior findings. Furthermore, the overall CUE in the YRB increased at a rate of 0.001, potentially linked to the impacts of climate change and anthropogenic vegetation restoration efforts. As shown in the study by Du et al. (2021), anthropogenic vegetation restoration not only boosts the capacity of ecosystems to sequester carbon but also heightens their carbon emission capacity. Taken together, the widespread increases in CWUE metrics indicate an improvement in ecosystem carbon sequestration efficiency in the YRB, driven by climate change and ecological engineering. This trend reflects a shift toward more water-efficient carbon assimilation by vegetation, particularly in restored areas.

Our research indicates that, from 1982 to 2018, the CWUE in the YRB demonstrated high sustainability, following a clear and sustainable growth pattern. This suggests that the current CWUE in the YRB is on a favorable developmental trajectory, with promising prospects for the future. Within the YRB, the WUE_GPP_ exhibits high stability in the northwestern part of the research area, whereas it shows substantial fluctuations in the southwestern region (Xu et al., 2023). Throughout the study period, both WUE_NPP_ and WUE_GPP_ in the YRB were characterized primarily by moderate fluctuations, while CUE exhibited low fluctuations. This indicates that the overall stability of CWUE in the YRB from 1982 to 2018 was favorable. This spatial pattern is shaped by distinct environmental drivers: precipitation dominance in the arid northwest results in higher stability, whereas multi-factor regulation in the southwest leads to greater variability. The widespread occurrence of high stability highlights the resilience of the basin ecosystem (Tian et al., 2020).

### 5.3 Impact of driving factors on CWUE

From 1979 to 2020, global precipitation exhibited a significant upward tendency. Additionally, the spatiotemporal variations of precipitation in the YRB consistent with the findings of this study (Gu and Adler, 2023), precipitation exerts a relatively strong negative effect on both WUE_NPP_ and WUE_GPP_. Between 2000 and 2018, LAI in the YRB showed a clear upward trend, aligning with the spatiotemporal trends of LAI in the Loess Plateau of China from 1985 to 2015, as reported in previous studies (Cao et al., 2020). In most parts of the YRB, WUE_GPP_ increased with the rise in LAI, in agreement with prior findings (Dou et al., 2024). LAI is the most crucial factor influencing WUE_GPP_ (Luo et al., 2022), and it is also the most significant factor for WUE_NPP_ (Li et al., 2016). Further analysis of regional differences reveals that in the southeastern part of the basin (lower reaches), radiation and LAI predominantly exert positive effects on WUE_NPP_, whereas in the northwest, the effects are mainly negative. Notably, the positive influence of LAI on CUE is particularly pronounced across the entire YRB. Temperature and precipitation have considerable impacts on WUE_NPP_, affecting vegetation growth in distinct ways depending on vegetation types and environmental conditions (Wang et al., 2023). Variations in climatic factors influence the carbon and water cycling processes within terrestrial ecosystems. These fluctuations lead to changes in GPP and ET, directly causing substantial alterations in WUE_GPP_ (Li et al., 2021). Temperature exerts a relatively strong negative effect on WUE_GPP_ in the YRB, while its positive effects on WUE_NPP_ and CUE are comparatively pronounced. In the YRB, the dynamics of WUE_NPP_ are more strongly influenced by precipitation than by temperature, a finding that is generally consistent with the research conducted by Gang et al. (2016). Consequently, anthropogenic vegetation restoration is likely to enhance ecosystem water use efficiency (Du et al., 2021). Temperature and precipitation were identified as the dominant drivers of the spatiotemporal variations in CUE. CUE decreased linearly with increased precipitation, consistent with the results of the study by Chen and Yu (2019). Higher temperatures may lead to an increase in CUE, with the impact of temperature varying across different ecosystems (Zeng et al., 2023).

### 5.4 Practical implications and limitations of the findings

The findings of this research explored the nonlinear spatiotemporal variation trends and patterns of CWUE changes in the YRB ecosystem. By combining the EEMD model, the optimized XGBoost model, and the SHAP model, the spatial heterogeneity of the key factors driving its spatiotemporal variation was revealed. The results of this study contribute to a deeper understanding of the carbon-water coupling process within the YRB ecosystem and provide a novel theoretical basis for ecological restoration, water resource management, and the achievement of the “double-carbon” goal, particularly in relation to the carbon-water use efficiency of the YRB ecosystem. However, several limitations exist in this study. Firstly, the GLASS data products used in this research still contain uncertainties and accuracy limitations, meaning that the processing results may be affected by some degree of error. Secondly, this study found that from 1982 to 2018, the CWUE in the YRB exhibited fluctuating growth. During this period, WUE_NPP_ and WUE_GPP_ primarily demonstrated monotonically increasing trends, while CUE mainly showed a pattern of first decreasing and then increasing, indicating relatively stable and sustainable behavior. However, this study does not provide predictions for the future spatiotemporal variation of CWUE in the YRB. Future work will aim to forecast the spatiotemporal variation of CWUE in the YRB over the coming decades, providing a stronger scientific foundation for the management of water resource management and ecological conservation within the basin, and helping the region achieve sustainable development.

## 6 Conclusions

This study analyzes the spatial distribution, nonlinear spatiotemporal dynamics, and variation patterns of CWUE in the YRB from 1982 to 2018, leveraging multi-source remote sensing data. Additionally, an optimized XGBoost model was employed to explore of the driving mechanism of CWUE. The findings of the study are as follows: (1) In the YRB, the spatial distribution of CWUE shows higher values in the southeast and lower values in the northwest, with considerable spatial heterogeneity across different latitudes and longitudes. CWUE is mainly concentrated at altitudes between 1000 and 1500 meters. (2) Monotonically increasing variation patterns of WUE_NPP_ and WUE_GPP_ show a predominantly monotonic increase over a large portion of the basin, covering approximately 42.44% and 41.97% of the total basin area respectively. In contrast, the dominant pattern for CUE is a decline followed by an increase, covering 42.51% of the total basin area. (3) In the YRB ecosystem, the leaf area index (LAI) emerged as the primary determinant of WUE_NPP_ and WUE_GPP_. Specifically, WUE_NPP_ and WUE_GPP_ both showed an upward trend in tandem with the increase in LAI. Furthermore, temperature was identified as the key driving factor for CUE within the YRB ecosystem. (4) The spatial variations of WUE_NPP_ and WUE_GPP_ are highly dependent on LAI, which LAI plays a dominant role in approximately 42.80% and 45.35% of the study area, particularly in the southern part of the YRB. However, for CUE, temperature is the primary contributing factor.

## Data Availability

The original contributions presented in the study are included in the article/supplementary material, further inquiries can be directed to the corresponding author/s.
